# Cognitive Physiology in Healthy Young Adults: Links to Ciliary Neurotrophic Factor and Retinal Biomarkers

**DOI:** 10.3390/diagnostics16183066

**Published:** 2026-09-21

**Authors:** Sarah Al-Mazidi, Laila Al-Ayadhi

**Affiliations:** 1 College of Medicine, Anatomy and Physiology Department, Imam Mohammad Ibn Saud Islamic University, Riyadh 11564, Saudi Arabia; 2 Autism Research and Treatment Center, King Saud University, Riyadh 11461, Saudi Arabia; 3 College of Medicine, Physiology Department, King Saud University, Riyadh 11451, Saudi Arabia

**Keywords:** cognitive impairment, OCT, optical coherence tomography, retina, CNTF

## Abstract

**Background:** Ciliary neurotrophic factor (CNTF) is a neurotrophic cytokine involved in neuronal survival, synaptic plasticity, and retinal ganglion cell health, and it correlates with different stages of cognitive impairment. Mild cognitive impairment (MCI) is difficult to diagnose, as these patients perform normally on standardized cognitive tests. It is important to diagnose patients with MCI early to prevent its progression and to maintain their quality of life. Alterations in retinal morphology mirror central nervous system pathology, including cognitive impairment. **Objective:** This cross-sectional study investigates the association between retinal structural changes and plasma CNTF levels and assesses their possible clinical value for screening early cognitive impairment. **Methods:** Retinal morphology was assessed by optical coherence tomography (OCT), and plasma CNTF was measured by enzyme-linked immunosorbent assay (ELISA) in 69 healthy participants. We correlated retinal biomarkers and CNTF levels with cognitive function as measured by the Montreal Cognitive Assessment (MoCA). **Results:** CNTF levels were significantly higher in the low MoCA group (median = 287.6, IQR: 235.90–359.7) compared to the normal cognition group (median = 185.90, IQR: 162.1–277.5) (*p* < 0.001). Average central thickness and retinal nerve fiber layer (RNFL) were positively associated with CNTF (*p* < 0.05). **Conclusions:** Our results indicate that CNTF is negatively associated with cognition and positively correlated with RNFL and average central thickness. Screening for MCI with non-invasive OCT imaging and plasma CNTF may aid in the early detection of cognitive impairment. Future pharmacological interventions with CNTF analogs may preserve or even restore cognitive function.

## 1. Introduction

Ciliary neurotrophic factor (CNTF) is a neurotrophin and a major member of the interleukin-6 (IL-6) family of cytokines. It is highly expressed in the central nervous system and retina and has a crucial neuroprotective role [1]. Patients with intellectual disabilities have significantly increased levels of CNTF as a physiological neuroprotective mechanism to support and reverse neural injury or degeneration [2]. Evidence in rats suggests that targeting CNTF can reverse neurotoxicity- and neuroinflammation-induced intracerebral neurobiological changes and improve intellectual abilities [3]. CNTF’s neuroprotective effects are mediated via the CNTF/JAK/STAT pathway, which may enhance brain-derived neurotrophic factor (BDNF) expression, a major regulator of neurogenesis, synaptogenesis, and synaptic plasticity [4].

Ciliary neurotrophic factor is a potent survival factor for retinal ganglion cells, photoreceptors, and cortical neurons, and protects against metabolic stress and injury [1]. Since the retina is considered an extension of the brain’s microstructure, changes in the retina can mirror systemic cognitive decline [5]. Ciliary neurotrophic factor delivered via encapsulated-cell intraocular implants improved retinal vascularization and macular thickness in a dose-dependent manner in patients with macular degeneration [6]. Studies reported that retinal morphology reflects cognitive impairment, specifically reduced macular thickness, and that the degree of thinning of the retinal nerve fiber layer is associated with the severity of cognitive impairment [5,7,8].

Cognitive physiology depends on a healthy neural environment, which can be affected by multiple factors. Cognitive dysfunction may result from neurological disturbances in which the patient presents with multiple symptoms alongside cognitive dysfunction, such as tumors, trauma, or infection [9]. Clinicians can diagnose this type of cognitive disorder using neuroimaging or cerebrospinal fluid analysis. Neurodegenerative cognitive impairment, such as dementia and Alzheimer’s disease (AD), is one of the major types of cognitive disorders that usually manifests only cognitive decline symptoms, mostly related to old age. Clinicians can diagnose this type of cognitive dysfunction using neurocognitive batteries and confirm it by measuring β-amyloid in cerebrospinal fluid and via Positron Emission Tomography (PET) scan [10].

Mild cognitive impairment (MCI) is the most common and challenging type of cognitive disorder, as individuals with MCI are overall healthy and score within the normal range on standardized cognitive tests. There are two types of MCI: amnestic MCI, which affects memory domains, such as in early stages of Alzheimer’s disease, and non-amnestic MCI, in which memory is spared. Both MCI types can affect a single cognitive domain or multiple domains, which determines the severity and progression of the cognitive impairment [11]. Beyond symptomatic differences between the two MCI types, structural differences also exist. Patients with amnestic MCI have decreased hippocampal, amygdala, and entorhinal cortical volumes compared to those with non-amnestic MCI [12]. These individuals with non-amnestic MCI have subtle cognitive complaints, but they can have serious safety implications that impact their quality of life. Diagnosis is therefore difficult, but early diagnosis is essential to prevent further cognitive decline. Studying biomarkers such as CNTF could support pharmacological therapy aimed at preventing cognitive decline and maintaining or even improving cognition in individuals with MCI. Continuous delivery of CNTF reduced β-amyloid synaptic damage, a hallmark peptide of dementia, and preserved memory in Alzheimer’s disease (AD) [13]. Also, higher levels of CNTF were found in the CSF of patients with cognitive impairment, and higher levels were associated with slower progression of cognitive impairment in patients with mild cognitive impairment [14].

Since the retina acts as a gateway to brain health, examining it would aid in screening and diagnosing cognitive impairment. Retinal examination can be performed using a simple imaging method known as Optical Coherence Tomography (OCT). Optical Coherence Tomography is a non-invasive imaging modality that captures retinal layers and nerve fibers. It is a reliable and available tool with no radiation risk. OCT has been used to detect degenerative retinal changes associated with cognitive decline in AD patients in multiple studies, making it a potential biomarker for screening for cognitive impairment [15].

While clinical trials have investigated the therapeutic potential of CNTF for retinal degenerations, its association with circulating neurotrophins and early cognitive physiology remains poorly understood. This study examines the associations among plasma CNTF levels, retinal layer thicknesses, and cognitive performance in a healthy young cohort to evaluate CNTF’s potential as an ocular-cognitive biomarker. 

## 2. Methods

### 2.1. Study Population

We recruited 69 healthy male and female volunteers aged 18 to 26 years from Applied Medical Sciences clinics at King Saud University who attended for routine eye examinations. We excluded participants with chronic systemic diseases, autoimmune diseases, systemic infection, regular use of medication, recent corticosteroid course, history of active ophthalmic pathology, high refractive errors, or neurological/psychiatric conditions. Approval of the Institutional Review Board was obtained (ID: E-23-8264), reference number 24/0008/IRB-A, following the Declaration of Helsinki, and all participants signed written informed consent. All participants could withdraw from the study at any time without consequences for their care at the clinic. Following consent, participants completed a survey collecting demographic information (age, gender, education level, place of origin) as well as past and current medical and medication history.

### 2.2. Neurocognitive Profiling

The cognitive screening was performed using the Arabic version of the Montreal Cognitive Assessment (MoCA). The MoCA assessment is a rapid screening instrument for mild cognitive impairment (MCI). It is a valid screening tool for MCI with 90% sensitivity [16]. It starts with simple tasks such as naming, tapping test, time and date orientation, and escalates to more complex executive tasks such as subtraction, drawing, and delayed recall. The test takes an average of 10 min and is performed by a certified health practitioner. The examiner explains the entire Montreal Cognitive Assessment test before starting. Each domain has a set time to complete, which also affects MoCA scoring. Participants were encouraged to ask questions, which the examiner answered clearly. The test starts when the participant is ready in a quiet room with comfortable seating. The variety of tests would allow the examiner to distinguish between severe cognitive dysfunction and mild cognitive impairment across seven distinct domains: visuospatial, naming, attention, language, abstraction, delayed recall, and orientation [17]. Each domain has a different score and a different level of complexity. Based on standard scoring, we grouped participants into either a normal cognitive group (scores 26 or higher) or a lower MoCA performance group (scores 18–25).

### 2.3. Optical Coherence Tomography (OCT)

We performed retinal structural profiling using standardized acquisition protocols with the Cirrus HD-OCT 6000 (Carl Zeiss Meditec, Dublin, CA, USA). Macular assessment was performed by displaying macular volume and central subfield thickness results according to the Early Treatment Diabetic Retinopathy Study (ETDRS) grid using a 512 × 128 macular cube protocol. Optic nerve assessment was performed by measuring average and quadrant-specific peripapillary RNFL thickness with a 200 × 200 optic disc cube protocol. We correlated macular volume and thickness, retinal nerve fiber layer thickness, and MoCA test domains with plasma CNTF levels in our study population. We defined total retinal thickness as the distance between the internal limiting membrane (ILM) and the retinal pigment epithelium (RPE).

### 2.4. Biochemical Analysis of Plasma CNTF

We collected 5 mL of peripheral blood from all participants into standard EDTA-coated tubes to prevent coagulation. We then centrifuged blood samples at 3000× *g* for 10 min to separate plasma. We aliquoted plasma into sterile cryovials and stored it at −80 °C until ELISA (Enzyme-Linked Immunosorbent Assay) analysis. We performed duplicate analyses of reference standards and plasma samples on a microplate using a commercial ELISA kit (MyBioSource Inc., San Diego, CA, USA, catalog number MBS2700772) according to the manufacturer’s instructions. This double-coated antibody sandwich ELISA has a manufacturer-reported detection range of 7.8 to 500 pg/mL and a sensitivity of <3.2 pg/mL. Briefly, wells were coated with a monoclonal antibody against ciliary neurotrophic factor (CNTF) as a capture reagent. Following incubation, unbound proteins were washed extensively. Target concentrations were quantified by measuring optical density and interpolating against a standard curve.

### 2.5. Statistical Analysis

Data were analyzed using the Statistical Package for the Social Sciences (SPSS 21.0 for Windows; SPSS, Chicago, IL, USA). We assessed data distribution and normality using the Shapiro–Wilk test. We expressed normally distributed data as mean ± standard deviation (SD), and non-normally distributed data as median with interquartile range (IQR). We evaluated associations using Pearson’s correlation coefficient for normally distributed variables and Kendall’s tau-b and Spearman’s rho for non-normally distributed data. We assessed group comparisons using the Mann–Whitney U test for non-normally distributed variables, testing the association between OCT-derived structural retinal measurements and plasma CNTF levels in individuals with normal cognition compared with those with lower MoCA performance, as defined by MoCA scores. *p*-values less than 0.05 were considered statistically significant. A post-hoc power analysis was conducted for the study primary findings (the difference in plasma eleven between the normal cognition and low mocha groups) the observed effect size (Cohen’s d = 0.93) corresponded to 97% achieved power at the study sample size *n* = 69 which is exceeding the conventional 80% threshold for adequate power. 

## 3. Results

### 3.1. Demographics and Cognitive Subgroups 

The study enrolled 69 healthy participants with a mean age of 22.88 ± 3.95 years, 38 males and 31 females. Participants had similar education levels and cultural backgrounds. The average MoCA score was 26.49 ± 2.37. Participants were divided into two groups: the normal cognition group (36 participants; mean MoCA score, 28.29 ± 1.17) and the lower MoCA performance group (33 participants; mean MoCA score, 24.5 ± 1.62) (Table 1).

### 3.2. Optical Coherence Tomography Analysis

Retinal morphology was assessed using Optical Coherence Tomography (OCT). The average retinal thickness, central thickness, macular volume, and retinal nerve fiber layer are demonstrated in Table 1.

**Figure 1:**
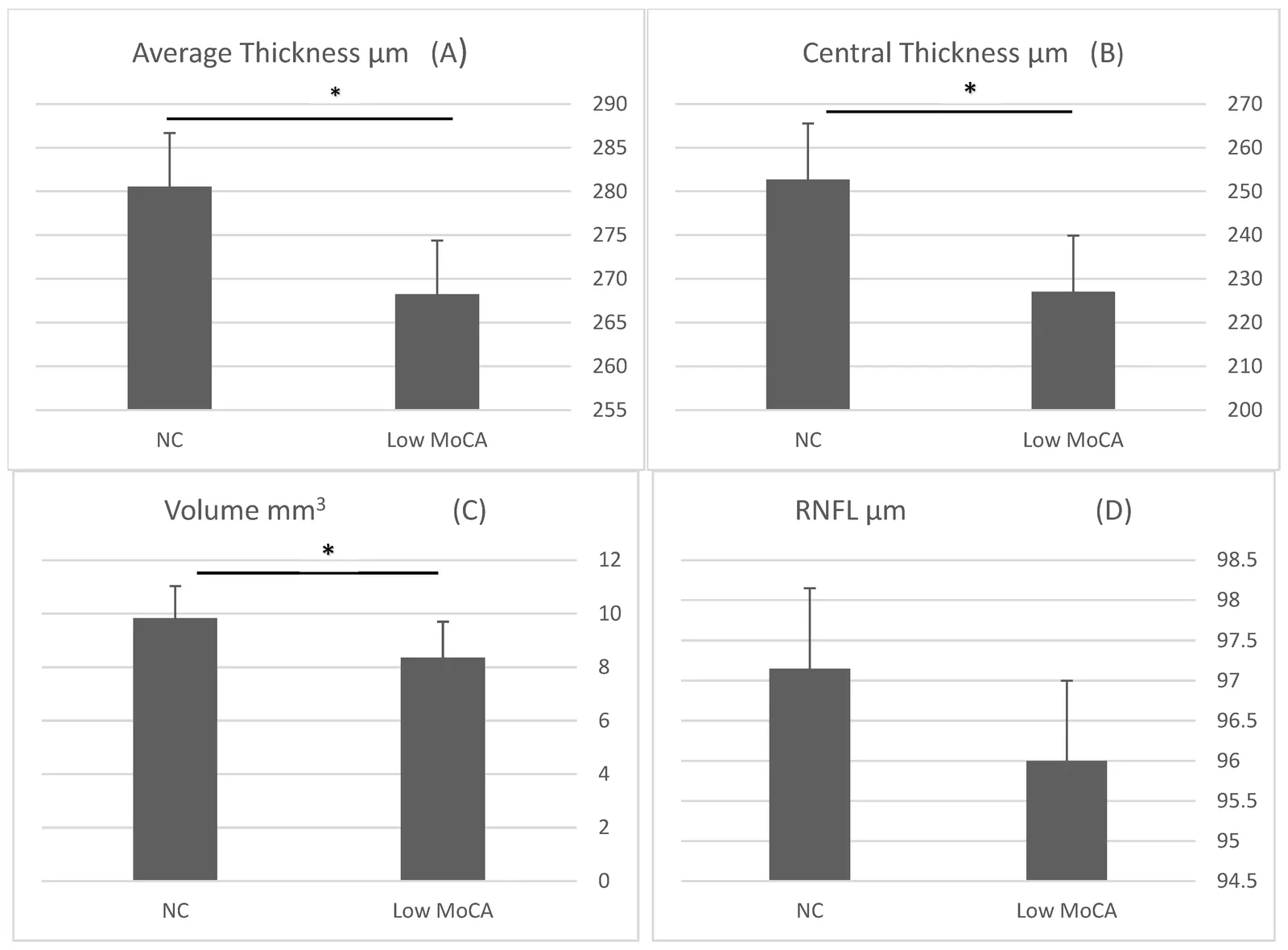
(**A**–**D**): Significant correlation between MoCA scores and OCT measurements. The lower MoCA performance group had significantly thinner macular layers (*p* < 0.005), reduced retinal volume (*p* < 0.001), thinner central thickness (*p* < 0.05), and thinner RNFL than the normal cognition (NC) group. Data were significant at *p* < 0.05 *.

The lower MoCA performance group exhibited significantly reduced average macular thickness (Figure 1A), central thickness (Figure 1B), and volume (Figure 1C). Retinal nerve fiber layer (RNFL) thickness was also thinner in the lower MoCA performance group than in the normal cognition group (Figure 1D).

### 3.3. Plasma CNTF Correlates

**Figure 2:**
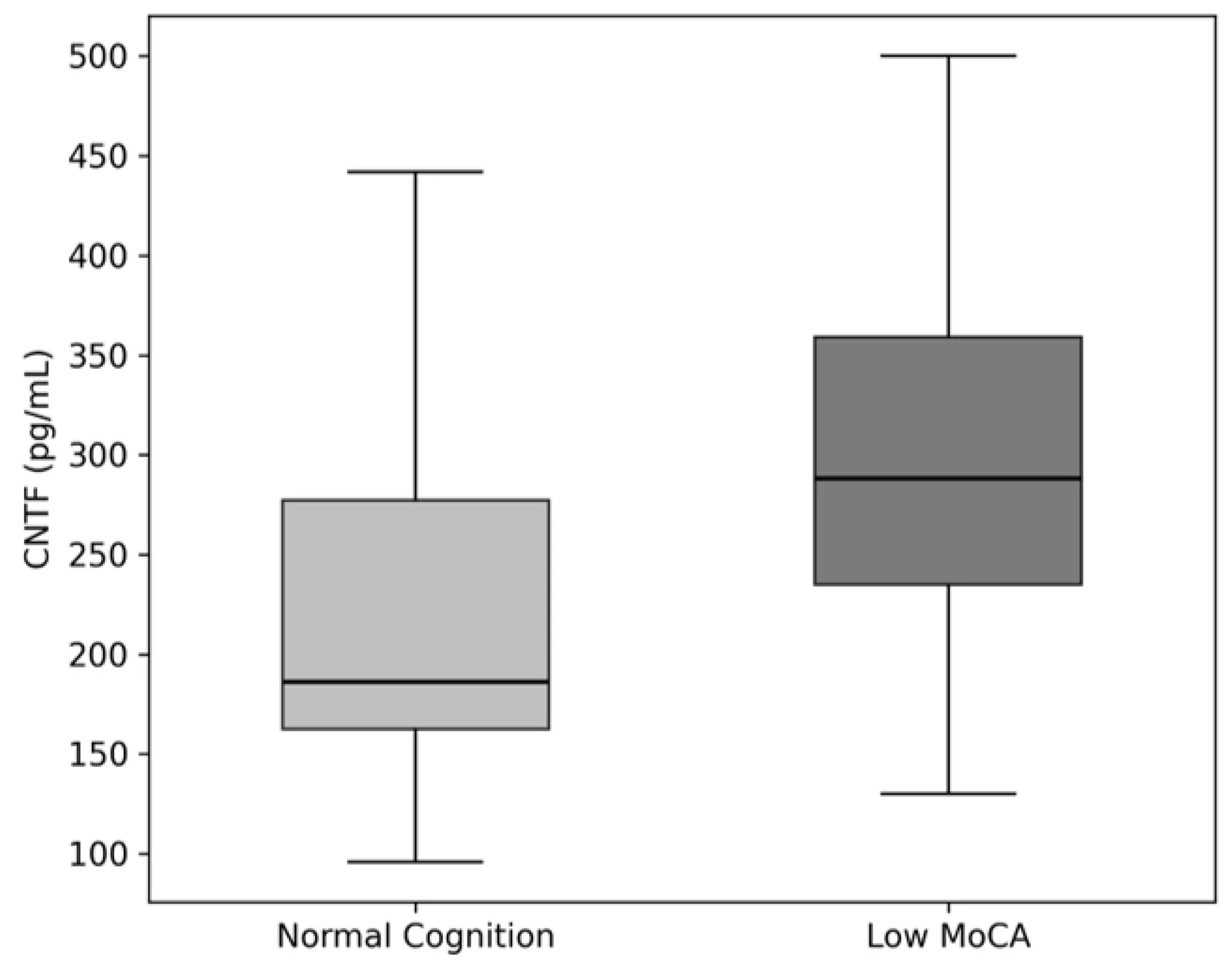
This figure shows a significant increase in CNTF levels in the low MoCA group compared with the normal cognition groups (Mann–Whitney U = 289.0, *p* < 0.001).

**Figure 3:**
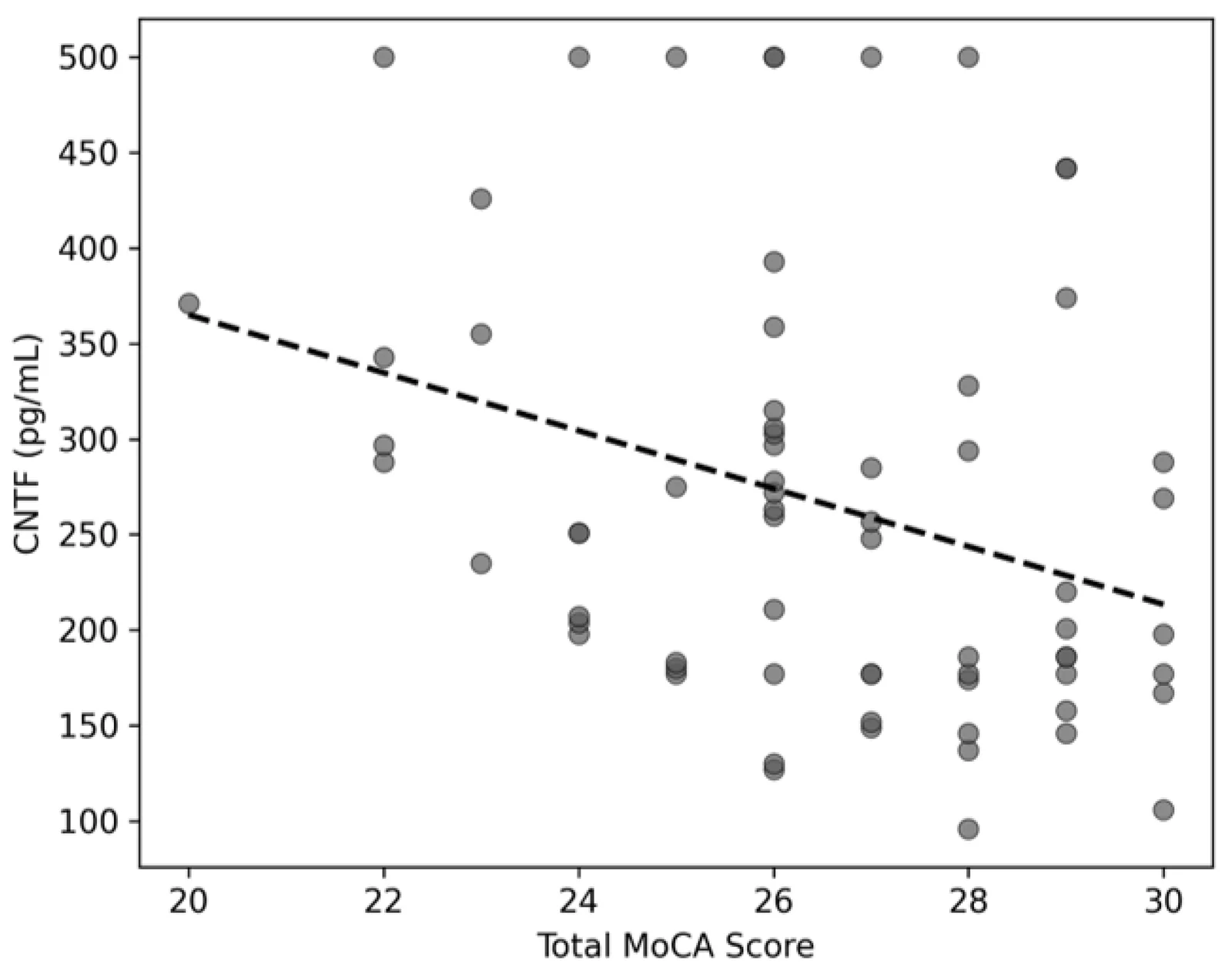
Significant association between higher levels of CNTF and lower levels of MoCA scores (Spearman’s *p* = 0.003).

We measured plasma CNTF levels in all participants and reported them as median and interquartile range (IQR). CNTF levels were significantly higher in the low MoCA group (median = 287.6, IQR: 235.90–359.7) compared to the normal cognition group (median = 185.90, IQR: 162.1–277.5), as determined by the Mann–Whitney U test (U= 289.0, *p* < 0.001) Figure 2. CNTF was also significantly related with MoCA scores. Figure 3 shows that higher CNTF scores were associated with lower MoCA scores.

We then compared CNTF levels with each MoCA domain, as shown in Table 2. Plasma CNTF levels increased across all MoCA domains in the lower MoCA performance group compared with the normal cognition group, and the increase was significant in the attention domain (*p* < 0.05).

### 3.4. Structure of the Retinal Layer Related to Plasma CNTF Levels

**Figure 4:**
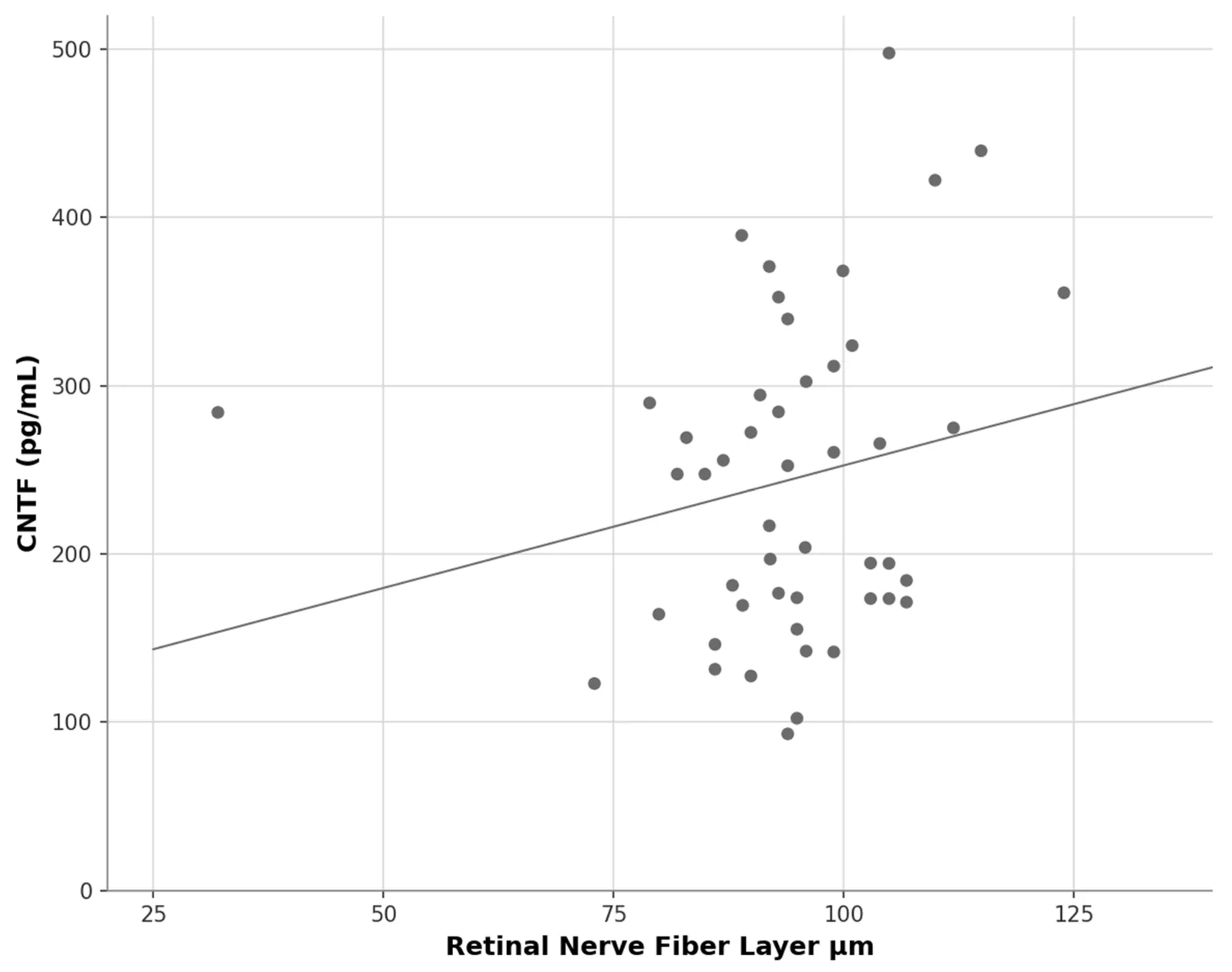
Positive association between CNTF and RNFL (*p* < 0.05) (*n* = 96).

The retinal nerve fiber layer was significantly associated with CNTF; RNFL was positively associated with CNTF (Figure 4), and this was significant in the low MoCA group (*p* < 0.05). Average central thickness was also positively associated with CNTF levels (*p* < 0.05), but this correlation was not significant for macular volume.

## 4. Discussion

This study demonstrates that Ciliary Neurotrophic Factor (CNTF) may be a biomarker of cognitive function. A key finding is the positive association between plasma CNTF and OCT measures of retinal nerve fiber layer and average central thickness, supporting neurotrophin’s protective role in the central nervous system and its extensions, such as the retina. Low CNTF levels are associated with reduced RNFL and average central thickness in patients with low cognitive function [18]. However, this is not limited to cognitive disease; it also includes different eye diseases, such as glaucoma, diabetic retinopathy, retinitis pigmentosa, and dry age-related macular degeneration (AMD), all of which are associated with reduced CNTF in the retina and thinning of the RNFL and the macula [6,19,20]. Thus, CNTF levels indicate overall neuronal health in the central nervous system and its extensions, including the retina [21].

Ciliary neurotrophic factors promote the survival of retinal ganglion cells and photoreceptors, protecting them from degenerative changes and thinning [1]. In early stages of neurodegenerative disease, mild retinal disease, nerve injury, and stroke, there is an increased expression of CNTF as a rescue mechanism, unlike chronic neurodegenerative diseases such as Alzheimer’s disease, schizophrenia, autism spectrum disorder, and advanced retinal disease, where CNTF was found to be low, which supports the notion that CNTF plays a neuroprotective role in the acute and mild stages of central nervous system disorders [4,22,23]. This aligns with our findings; individuals with lower MoCA performance exhibit elevated plasma CNTF levels, concurrent with thinning of the RNFL and macular thickness, possibly reflecting an active, upregulated neuroinflammatory and protective distress response to early subcortical neurodegeneration. However, intra-group analysis showed a positive association between CNTF and retinal structure, indicating that macular and RNFL thickness increase with increasing CNTF levels, validating CNTF’s role as a vital, active neuroprotective agent against progressive structural atrophy in individuals with lower MoCA performance. In healthy individuals, thicker retinal structure is not associated with CNTF activity, because CNTF is actively expressed in neural dysfunction, which explains the lower CNTF levels in the normal cognitive group compared to the lower MoCA performance group.

Treating degenerating retinas with CNTF improves retinal structure and restores metabolic function by promoting aerobic glycolysis, thereby improving neuronal viability [1]. Recently, this led to the introduction of Revakinagene taroretcel-lwey, the first FDA-approved Encapsulated Cell Technology that continuously releases recombinant CNTF to treat Macular Telangiectasia Type 2, a retinal condition associated with retinal thinning and reduced retinal CNTF levels, improving macular health on OCT by 30% to 56% over 24 months [24]. The therapeutic effect of CNTF on macular OCT supports the finding that higher serum CNTF levels positively correlate with macular thickness, further emphasizing CNTF’s potent neuroprotective effects, which support neuronal survival and slow degenerative processes. Other studies have shown a similar effect in retinitis pigmentosa and dry AMD [25,26].

In our study, we found a significant negative association between CNTF and cognitive performance, with plasma CNTF levels higher in participants with lower MoCA performance than in normal controls. This increase likely represents an endogenous, early neuroprotective response. In response to early, subclinical neural stress, the body may upregulate CNTF to stimulate neuroplasticity and protect vulnerable neuronal pathways. This response may be mediated by downstream pathways, such as Brain-Derived Neurotrophic Factor (BDNF), which support neuronal survival and cognitive function [27]. CNTF treatment has a neuroprotective effect, promotes plasticity, and may improve cognitive impairment by increasing BDNF levels. Brain-derived neurotrophic factor is crucial for retinal ganglion cells and is a protective neurotrophin found in neurodegenerative diseases such as Glaucoma, Alzheimer’s disease, Parkinson’s disease, and Huntington’s disease [27,28]. The neuroprotective role of CNTF increases following nerve injury, as we speculate occurred in our study, in which CNTF increased in individuals with lower MoCA performance [29].

Individuals with mild cognitive impairment can maintain independent daily activities. However, diagnosing mild cognitive impairment as early as possible is crucial to prevent deterioration and preserve neuronal health, thereby improving quality of life. Treatments can range from dietary supplementation to pharmacological interventions to maintain cognition and, in some cases, reverse cognitive impairment. Simple lifestyle modifications, such as a diet rich in Omega-3 fatty acids, fasting, carbohydrate restriction, and an exercise regimen, can improve the production of neuroprotective factors in early cognitive impairment [30]. Combining treatments that include both CNTF and BDNF provides superior neuroprotection [31]. These and many other treatment options are available or are currently under clinical trials, which can support and reverse neurodegeneration in mild cognitive impairment, ensuring independence and reducing the probability of harm to self and their families. The main issue remains in diagnosing mild cognitive impairment, as these individuals have normal cognitive performance and show minor cognitive impairment in some domains, such as recall, naming, memory, and attention, while preserving executive cognitive functions [32]. Identifying biomarkers would support early diagnosis and enable early intervention, thereby preserving or reversing neurodegeneration and improving quality of life.

The current study showed that average central thickness and the retinal nerve fiber layer are potential biomarkers of cognitive physiology, as measured by OCT. It might be used for screening and ongoing monitoring of cognitive impairment. The OCT imaging procedure requires multiple images, fixation, and following the examiner’s instructions, which were not an issue for our participants with lower MoCA performance, unlike patients with severe dementia, in whom OCT is technically challenging because these patients may not be cooperative and may produce many motion artifacts, which might be a disadvantage of OCT assessment in patients with advanced cognitive decline. In addition, CNTF is a potential cognitive function biomarker and might serve as a complementary screening method for cognitive performance, as supported by previous reports. Integrating these findings with artificial intelligence (AI) models trained on extensive ocular datasets may facilitate early prediction of cognitive decline, thereby enabling timely lifestyle modifications and cognitive training before irreversible damage occurs.

### Limitations

This study recruited patients attending the optometry clinic for routine eye examinations, which may introduce selection bias that we cannot precisely quantify with the available data. Therefore, our data may not generalize to broader or more diverse populations. An adequately powered, ideally multi-site or population-based study is warranted to confirm the retina–CNTF relationship with cognition. As a cross-sectional study, this design does not permit inference of a causal relationship between CNTF, OCT measures, and cognitive status.

## 5. Conclusions

Ciliary Neurotrophic Factor is positively associated with average central retinal thickness and RNFL in individuals with lower MoCA performance, suggesting a possible clinical value of CNTF and OCT as a screening tool for MCI. Combining OCT measurements of macular thickness and retinal nerve fiber layer thickness with peripheral CNTF levels might improve the mild cognitive impairment screening protocol. Detecting structural and biochemical changes during the early phase provides a critical opportunity for early intervention and improving quality of life.

## Figures and Tables

**Table 1:** Comparison of study data between normal cognition and low MoCA groups.

Measured Outcomes	NC	Low MoCA	Total
Age	22.49 ± 3.76	22.91 ± 3.53	22.88 ± 3.95
MoCA	28.29 ± 1.17	24.5 ± 1.62 *	26.49 ± 2.37
Average Thickness µm	280.93 ± 12.44	268.27 ± 14.38 *	275.44 ± 14.5
Central Thickness µm	252.84 ± 25.45	227.09 ± 35.46 *	242.05 ± 32.1
Volume mm^3^	9.80 ± 1.08	8.33 ± 1.22 *	9.19 ± 1.3
Retinal nerve fiber layer µm	96.44 ± 8.81	96 ± 10.66	96.21 ± 9.4

NC = normal cognition, MoCA = Montreal Cognitive Assessment, *p* value significant at (*p* < 0.05) *.

**Table 2:** Demonstration of the correlation between MoCA domains and plasma levels of CNTF (median, IQR).

	CNTF pg/mL	
MoCA Domains	Normal Cognition Median (IQR)	Lower MoCA Median (IQR)
Visuospatial	220.0 (177.0–294.0)	249.5 (177.8–352.0)
Attention	186.0 (174.0–288.0)	269.0 (199.5–358.0)
Language	229.0 (177.0–289.5)	248.0 (177.0–400.0)
Abstraction	248.0 (177.0–328.0)	272.5 (223.2–321.8)
Delayed recall	198.0 (174.0–288.0)	261.5 (180.8–339.2)
Orientation	235.0 (177.0–306.0)	363.0 (310.5–403.2)

## Data Availability

The raw data supporting the conclusions of this article will be made available by the authors on request.
